# The DEPFET Sensor-Amplifier Structure: A Method to Beat 1/f Noise and Reach Sub-Electron Noise in Pixel Detectors

**DOI:** 10.3390/s16050608

**Published:** 2016-04-28

**Authors:** Gerhard Lutz, Matteo Porro, Stefan Aschauer, Stefan Wölfel, Lothar Strüder

**Affiliations:** 1 PNSENSOR GmbH, München D-81739, Germany; gerhard.lutz@pnsensor.de (G.L.); stefan.aschauer@pnsensor.de (S.A.); 2 European X-ray Free-Electron Laser Facility GmbH, Hamburg D-22761, Germany; matteo.porro@xfel.eu; 3 Gründeläckerstr. 28, Dormitz D-91077, Germany; stefan.woelfel@steppke.de; 4 Experimental Physics, University of Siegen, Walter-Flex-Str. 3, Siegen D-87068, Germany

**Keywords:** DEPFET, photon detection, sub-electron precision, charge measurement, pixel detector, X-ray spectroscopy

## Abstract

Depleted field effect transistors (DEPFET) are used to achieve very low noise signal charge readout with sub-electron measurement precision. This is accomplished by repeatedly reading an identical charge, thereby suppressing not only the white serial noise but also the usually constant 1/f noise. The repetitive non-destructive readout (RNDR) DEPFET is an ideal central element for an active pixel sensor (APS) pixel. The theory has been derived thoroughly and results have been verified on RNDR-DEPFET prototypes. A charge measurement precision of 0.18 electrons has been achieved. The device is well-suited for spectroscopic X-ray imaging and for optical photon counting in pixel sensors, even at high photon numbers in the same cell.

## 1. Introduction

The most prominent request for deep sub-electron noise performance arises from imaging, spectroscopy and photon counting in the visible domain. Either low light level applications or the constant shrinking of pixel sizes reduces the number of signal charges to be detected, and a read noise reduction is therefore required to obtain a decent signal-to-noise ratio.

Single photon detection in the wavelength range from 300 nm (E = 4 eV) to 1.100 nm (E = 1.1 eV) is of interest for many applications in science and industry. In the wavelength range under discussion (300 nm to 1.100 nm), every photon penetrating and converting in the silicon produces one electron-hole pair [1]. For photon energies above 4.3 eV the creation of two electron-hole pairs starts to emerge. In the case of near-infrared photons at 1100 nm (1.1 eV) the detector has to be sensitive over a deep sensor volume, e.g., 500 μm to achieve a quantum efficiency above 35% as the attenuation length for this wavelength is approximately 400 μm. At wavelengths in the ultra-violet region of 300 nm, the absorption length in silicon is only 8 nm, *i.e.*, the absorption of the photon happens very close to the radiation entrance window. Detectors covering this full bandwidth have to cope with a difference of the absorption depth of the photon in the sensor of a factor of 5000 while the quantum efficiency ideally has to be high and constant. 

The DEPFET detectors (depleted field effect transistor) perform close to the above experimental requirements. DEPFETs represent a sensor and amplifier structure simultaneously. The generated signal electrons are collected and confined in a potential minimum underneath the transistor gate, where the stored signal charges modulate the DEPFET current. This allows for fast gating, analog storage, repetitive non-destructive readout and the absence of reset noise. For the sake of simplicity we confine our study to silicon as a detector and electronics material.

To get a truly linear amplifier system for single photon counting, the readout noise of the sensor has to be significantly lower than 1 electron (rms), e.g., 0.2 electrons (rms). Floating gate amplifier systems have been studied in the past with some success (see e.g., [2]). The repetitive reading of the same charge package allows for reducing the 1/f noise contribution with approximately the square root of the number of readings for a given fixed signal processing time. 

We will first describe the DEPFET concept and basic operation including its use in X-ray sensor systems in Section 2. Section 3 will present the mathematical treatment of 1/f noise and briefly discuss the influence and limitations of other noise components. In Section 4 we describe the experimental verification with the help of test devices to check the models and to demonstrate the single photon resolution up to several hundred optical photons. The best noise performance achieved was 0.18 electrons (rms). A final outlook will be provided regarding how a DEPFET floating gate sensor-amplifier can be converted into an optical sensor system. 

## 2. The Depleted p-Channel MOSFET (DEPFET)—A Detector-Amplifier Structure

The DEPFET structure, invented in 1985 by Kemmer and Lutz [3], possesses unique properties that make it extremely useful as a radiation sensor, in particular as a basic cell of a pixel detector. The DEPFET combines the properties of sensors, amplifiers and signal charge storage and allows for non-destructive reading. A variety of DEPFET structures have been invented, with properties such as gateability and signal compression [4], macro-pixel DEPFETs (a combination of DEPFET and silicon drift detector [5,6]) and DEPFETs with intermediate signal charge storage [7]. DEPFETs with repetitive non-destructive readout (RNDR) that allow sub-electron charge measurement precision [8] are the focus of our paper.

**Figure 1:**
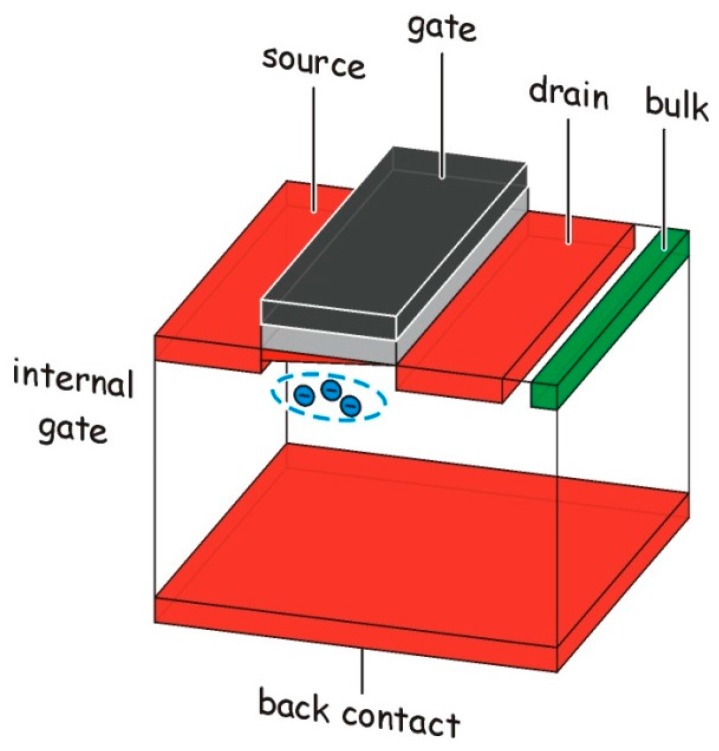
The concept of a DEPFET: The signal electrons are collected in a potential minimum (internal gate) located below the channel of a FET located on top of the fully depleted bulk.

Basically, the DEPFET (Figure 1) is a field effect transistor with the source (S), drain (D) and (external) gate (G) located on the front side of a wafer and a large area diode on the back used to fully deplete the bulk. With suitable doping, a potential minimum below the transistor channel is created. Signal charge created anywhere within the depleted bulk assembles in the potential minimum thereby creating mirror charges in the channel and increasing the transistor current. Due to the current steering function, the potential minimum is called the internal gate (IG). Furthermore, a DEPFET contains a device for removing all charge from the IG. With an empty internal gate, a base current I_0_ is flowing through the transistor. At the time a radiation signal is created, the conversion electrons will rapidly move towards the IG and increase the transistor current to I_1_. Emptying the IG restores the current to its original value I_0_. Either one of these current steps (rise or fall) is a measure for the signal charge.

The following physical properties contribute to the noise: thermal fluctuations of the distribution of charge carriers in the transistor channel are the source of white serial noise; thermal fluctuations of the leakage current in the detector volume that lead to parallel white noise; and capture and reemission of charge carriers within the transistor channel in defect locations close to the silicon-SiO_2_ interface. Trapping and de-trapping times are different for each individual trapping center. The superposition of many such locations results in a noise spectrum that is approximated by a 1/f (serial) spectrum.

**Figure 2:**
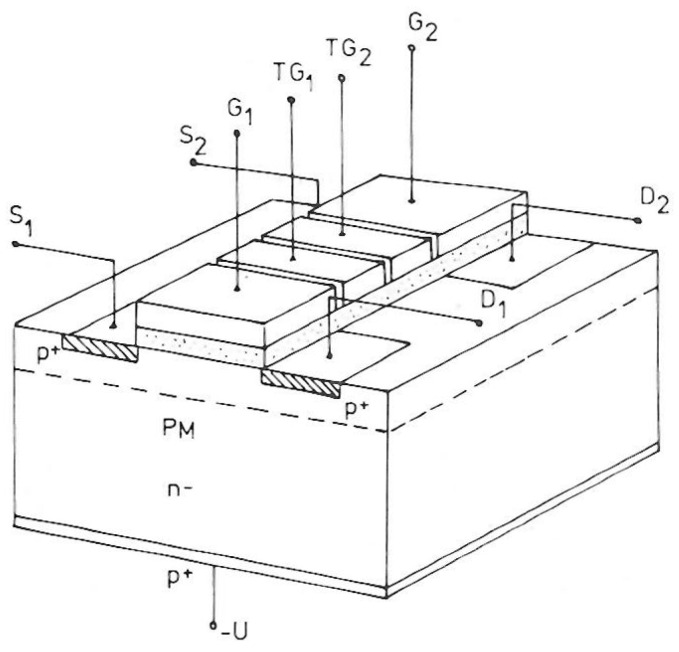
Ping-pong arrangement of DEPFETs in the original publication.

As mentioned before the charge is by design not destroyed during the readout process. To preserve the charge it is sufficient to shift the signal charge from the IG to a storage position and use the resulting current step caused by this operation as a measure of the signal charge. Shifting the charge back to the IG and out again results in a second measurement of the charge. Repeating this cycle n times results in a measurement improvement of approximately a factor sqrt(n). This holds not only for serial white noise but also for 1/f noise. The proposal for repetitive non-destructive readout (RNDR) was already contained in the original publication [3]. There, charge shifting was done between the Internal Gates of two closely spaced DEPFETs connected by a CCD-like transfer structure (Figure 2).

A mathematical correct treatment of noise and measurement precision will be given in the following section and the experimental results will be presented thereafter.

## 3. DEPFET Readout

In order to measure the signal charge collected in the internal gate of the DEPFET, it is necessary to perform two evaluations. We can assume that before the signal charge arrival, the internal gate is completely empty, since all the charge (both the signal charge of the previous measurement and the leakage current charge) has been removed by a clear pulse. The output of the DEPFET, *i.e.*, the drain current or the source voltage, is measured. This corresponds to evaluation of the baseline of the system, the output corresponding to the empty internal gate. Then, the signal charge is collected into the internal gate of the DEPFET and a second measurement of the device output is performed: the baseline + the signal are evaluated. The difference of the two evaluations (baseline only and baseline + signal) gives the information about the amount of the signal charge. Therefore, every complete measurement is always composed of two evaluations:

• Baseline
• Baseline + Signal

Since the signal arrival time is known, a time variant filter is used for the system readout. One common readout method in APS systems is correlated double sampling (CDS). In this scheme, two correlated samples of the voltage output of the device are taken and subtracted from each other, one sample corresponding to the baseline and one sample corresponding to the baseline + signal. Instead of taking only one sample for each single evaluation, an average of several samples can be taken, performing a multi-correlated double sampling (MCDS) [9]. In most cases this would improve the noise performance of the system. Another readout possibility is to integrate the output current of the device for a certain amount of time instead of sampling its voltage output. This would correspond to an ideal MCDS with an infinite number of samples. In this work we refer to such a current integrating filter that provides a triangular weighting function, *i.e.*, the optimum time-limited filter for white voltage noise. This is the dominant noise source at high speed. In the real case, a trapezoidal weighting function must be used. In fact, a flat-top is necessary to let the output of the DEPFET settle after transferring the charge and to reset some stages of the readout electronics. If the flat-top is relatively short with respect to the total length of the weighting function, the results reported in this paper are not considerably affected. Such a filter has already been successfully implemented in multi-channel readout ASICs for DEPFETs and pnCCDs [10,11].

The operation of subtracting the baseline from the baseline + signal evaluation, independently from the acquisition method (voltage sampling or current integration), results in a high-pass filter. The low-pass limitation is given by the bandwidth of the sampling circuit in the case of CDS or MCDS, and by the integrating process itself in the case of the device current evaluation. 

## 4. RNDR-DEPFET

**Figure 3:**
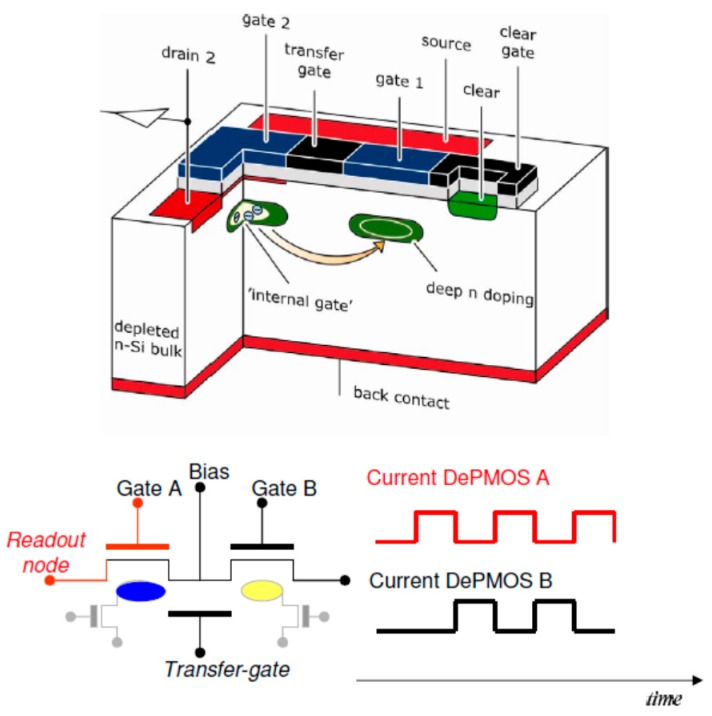
Simplified drawing of a section of a RNDR-DEPFET. The device is composed of two adjacent DEPFET structures with two independent internal gates. Thanks to a transfer gate, the signal charge can be transferred from the internal gate of one DEPFET to the internal gate of the other one. Moving the charge back and forth, it is possible to reproduce and read out the output signal arbitrary often. During operation, when the internal gate of one device is full, the internal gate of the other device is empty and vice versa. This is schematically represented by the plot of the output current of the two transistors. When one transistor has the maximum output current (the internal gate is full), the other transistor has zero signal output current. When the charge is then transferred, the situation is the opposite. For multiple readout it is possible to read out both the transistors or only one. If only one transistor is read out, the other one acts just as a storage device for the signal charge.

The RNDR-DEPFET device [12] is composed of two adjacent DEPFET structures, with two individual and insulated internal gates (Figure 3). 

The charge in the internal gate of one device can be transferred back and forth to the internal gate of the other device, thanks to one (or more) transfer gate(s). For a proper operation, when the internal gate of one device is full, the internal gate of the other one must be empty and vice versa. Moving the signal charge from one device to the other allows one to reproduce the DEPFET output signal arbitrarily often. In this way the signal charge can be read out non-destructively many times. The main limitation is given by the leakage current that fills the internal gate and spoils the original signal [12]. It is possible to read out the signal from both devices or to evaluate the output of only one device. In the last case the other device is used just as storage for the signal charge. For the sake of simplicity we studied a case in which we read out only one device. It is worth pointing out that, in order to read-read out the signal many times, it is necessary to transfer it from one DEPFET to the other. In fact, as already stated, every measurement is composed of two evaluations: baseline and signal + baseline. It follows that, if we want to read out n times the signal from one of the two devices of the RNDR-DEPFET, e.g., DEPFET (A) in Figure 3, we have to reproduce n times both the baseline, *i.e.*, the output corresponding to empty internal gate, and the signal+ baseline, *i.e.*, the output corresponding to the internal gate filled by signal charge. If we want to reproduce the baseline of transistor (A), without destroying the signal information, we have to move the signal charge into transistor (B), which acts, as already stated, as a charge storage device. Moving back the charge to transistor (A) the output corresponding to baseline + signal is then reproduced. This procedure can be repeated arbitrarily often. The RNDR procedure must not be confused with the MCDS. The MCDS, in fact, refers to the case in which a single baseline or signal + baseline evaluation is obtained by averaging a certain number of samples. The RNDR refers to the case in which the complete measurement procedure (subtraction of the baseline from the signal + baseline) is repeated several times. As already stated, this requires the reproduction both of the signal and of the baseline.

## 5. DEPFET Noise Analysis

### 5.1. Noise in Single Readout

In order to better appreciate the benefits of the repetitive non-destructive readout technique, it is useful to discuss the achievable noise performance of a traditional spectroscopic system performing a single readout.

**Figure 4:**
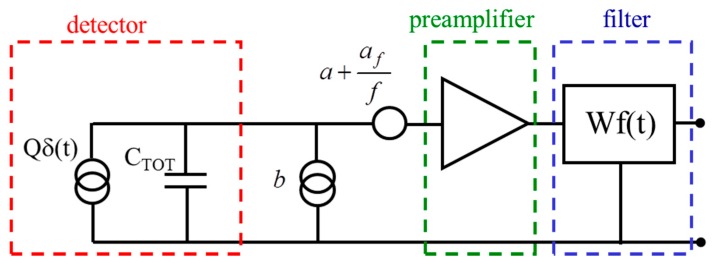
Schematic of a typical spectroscopic chain. The detector is modeled as a delta-like current source in parallel to a capacitance. The input referred noise sources are represented. In this work we consider only the series noise sources (white and 1/f) and a filter triangular weighting function Wf(t).

The spectroscopic chain can be represented as in Figure 4 and its associated equivalent noise charge can be expressed with the well-known formula [13]:

(1) $$ENC^{2}=\frac{a}{\tau }C_{TOT}^{2}A_{1}+2\pi a_{f}C_{TOT}^{2}A_{2}+b\tau A_{3}$$

The three terms of the ENC formula represent the main three noise contributions, *i.e.*, the series white, the series 1/f and the parallel white noise contributions.

• C_TOT_ is the equivalent input capacitance of the system.
• *a*, *a_f_* and *b* are the series white, the series 1/f and the parallel white physical noise sources, referred to the input.
• A_1_, A_2_ and A_3_ are the filter parameters. They depend on the shape of the weighting function Wf(t) implemented by the readout electronics.
• *τ* is the shaping time of the readout filter and is an expression of the time needed to perform one measurement.

**Figure 5:**
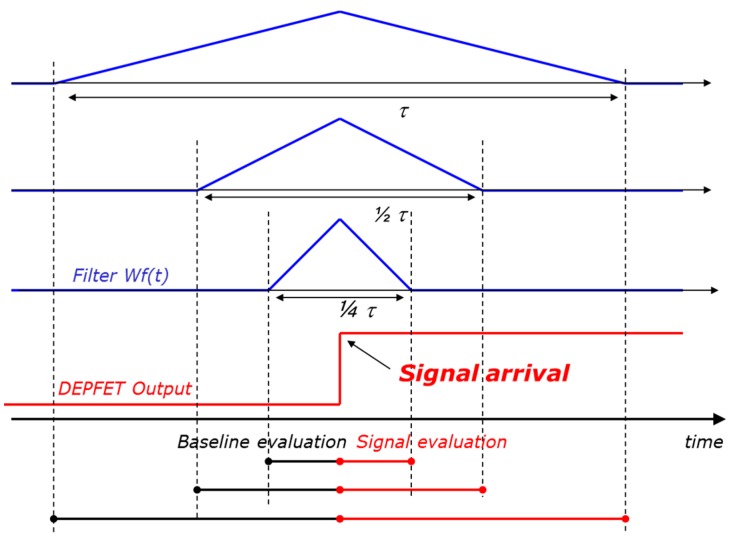
The same signal (red line) is processed by three triangular weighing functions with three different shaping times. The ENC component due to the white voltage noise goes down as the shaping time increases, while the ENC component related to the 1/f noise stays constant. Increasing the shaping time the ENC goes down up to the point in which it is dominated by the 1/f noise. A further increase of the shaping time would not provide any benefit.

In this work we focus on the series noise contributions (white and 1/f) and we neglect the effect of the leakage current, which can be minimized by cooling. For this reason the A_3_ coefficient is not considered in the following text. From Equation (1) it is evident the 1/f component of the ENC is independent from the shaping time of the system, but depends only on the physical 1/f noise source and on the type of filter, which determines the coefficient A_2_. This means that the term of the ENC related to the 1/f noise is independent from the time used to process one signal. In contrast, the ENC term due to the white voltage noise decreases as the shaping time increases. So, once the type of signal processing has been defined, e.g., a triangular weighting function, in order to increase the signal-to-noise ratio, it is possible to increase the shaping time of the filter. This is true as long as the measurement time is so large that the system becomes dominated by 1/f noise. At this point a further increase of the shaping time would not bring any benefit, since the 1/f noise contribution does not scale with τ. Figure 5 shows the same signal processed by three triangular weighting functions having three different time lengths (τ, ½τ and ¼τ). The change of the shaping time modifies only the white noise component of the ENC.

### 5.2. Noise in Repetitive Non-Destructive Readout

We can assume to fix the total measurement time τ_TOT_, e.g., we choose a τ_TOT_ for which the 1/f noise contribution is dominant. This means that the ENC would not significantly decrease for any τ > τ_TOT_. If we make one measurement exploiting the whole time interval τ_TOT_, thanks to Equation (1) and remembering that the ENC is obtained equating the Noise-to-Signal ratio (N/S) to one (see [13,14,15]), it is possible to write:

(2) $${\left(\frac{N}{S}\right)}_{\text{white},\;\text{single}}^{2}\propto A_{1}\frac{1}{\tau_{\text{TOT}}}$$

and

(3) $${\left(\frac{N}{S}\right)}_{1/f,\;\text{single}}^{2}\propto A_{2}$$

**Figure 6:**
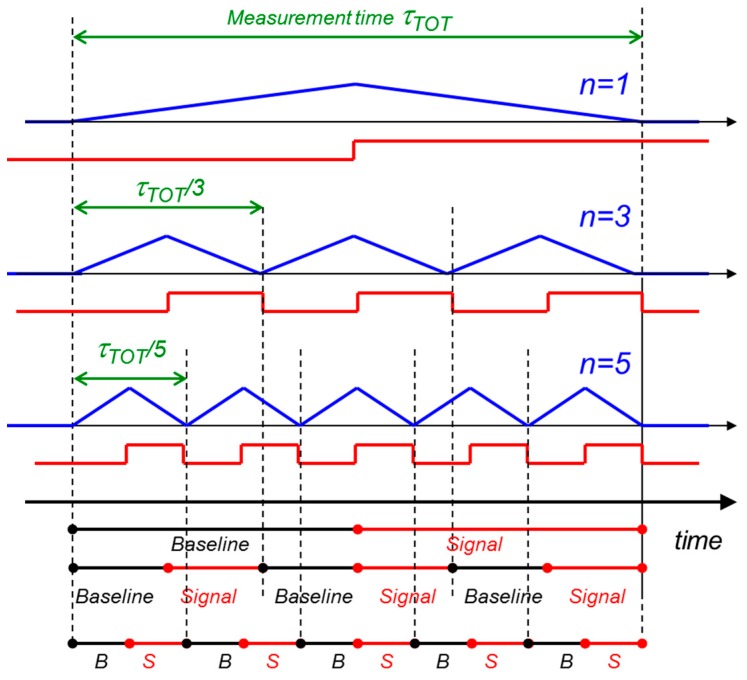
Schematic representation of the multiple non-destructive readout with fixed total measurement time τ_TOT_. In the upper drawing, only one readout of the signal (red line) is performed, exploiting the whole τ_TOT_. In the other two cases, the signal is reproduced and read out 3 and 5 times respectively. Since τ_TOT_ is fixed, the time available for each single measurement is τ_TOT_/3 and τ_TOT_/5. The reduction of the shaping time for each single measurement (look at the blue weighting functions) turns out in an increased r.m.s. value of the white noise of the individual measurements. The averaging effect of the n readouts (3 and 5 respectively) compensates this noise increment. Therefore the ENC component related to the white voltage noise does not change with the number of measurements in a fixed time interval. For the 1/f noise the situation is different. The r.m.s. value of noise of one measurement is independent from the measurement time. This means a single measurement of time length τ_TOT_, τ_TOT_/3 or τ_TOT_/5 would result in the same r.m.s. noise. In this case, the averaging effect of n measurements makes the ENC go down with approximately √n. In the figure only the positive step of the signal is measured, *i.e.*, the signal is measured only when it is injected into the internal gate. In theory it would be possible to measure the signal charge also when it is removed from the internal gate, evaluating the falling edge of the DEPFET output. This would increase of a factor two the number of measurements for a certain number of charge transfers.

We can then reproduce, within the time interval τ_TOT_, the signal n times. This can be done by moving the charge back and forth to the internal gate of one device of the RNDR-DEPFET structure and using the other device as charge storage. In this way it is possible to measure the signal n times and make an average of the measurements. We hypothesize that the signal we reproduce is always the same, *i.e.*, that the signal charge is not spoiled by leakage current electrons that can cumulate in the internal gate. Since τ_TOT_ is fixed, the time available for each single measurement is τ_TOT_/n, as shown in Figure 6.

Since the n measurements are almost independent, making an average the noise sums up quadratically, while the signal sums up linearly. Now let us consider the case of the white voltage noise. If we measure the signal n times within the same total time, the r.m.s. value of the noise of each single measurement is increased because the measurement time has been reduced by a factor of 1/n (see Figure 6). If we want to express the noise-to-signal ratio of the average of n measurements, we have to quadratically sum up the noise of the single measurements and linearly sum up the signal. If we assume that the amplitude of a single signal is normalized to one, then the amplitude of the sum of the n signals is just n. Therefore it is possible to write:

(4) $${\left(\frac{N}{S}\right)}_{\text{white},\;\text{multiple}}^{2}\propto \frac{n\left(A_{1}\frac{n}{\tau_{\text{TOT}}}\right)}{n^{2}}=A_{1}\frac{1}{\tau_{\text{TOT}}}$$

Comparing Equations (2) and (4) it is evident that the multiple readout has no effect on the ENC component due to the white voltage noise, when the measurement time is fixed. The benefit of averaging many readouts is compensated by the increase of the r.m.s. noise of the single measurements, due to the shorter shaping time. For the 1/f noise the situation is different, since in this case the r.m.s. value of the noise is independent from the measurement time. Therefore, a measurement performed in a time τ_TOT_ and a measurement performed in a time τ_TOT_/n lead to the same 1/f component of the ENC. This means that averaging n measurements with a shaping time τ_TOT_/n is better than performing only one measurement with the longest possible shaping time τ_TOT_. In fact the noise-to-signal for the 1/f noise, in the case of n measurements, can be expressed as:

$${\left(\frac{N}{S}\right)}_{1/f,\;\text{multiple}}^{2}\propto \frac{{\text{nA}}_{2}}{n^{2}}=\frac{A_{2}}{n}$$

This means that the ENC component related to the 1/f noise scales approximately with √n. Actually, to be precise, the (N/S) scales as:

$${\left(\frac{N}{S}\right)}_{1/f,\;\text{multiple}}^{2}\propto \frac{A_{2}}{n^{\alpha }}$$

where α is a coefficient very close to one. This is due to the fact that the noise of the different measurements is not completely uncorrelated. To make a rigorous calculation the reader can refer to [14,15,16].

In summary, when the total measurement time is fixed:

• ${\left(\frac{N}{S}\right)}_{\text{white}}^{2}$ is independent from the number of measurements n
• ${\left(\frac{N}{S}\right)}_{1/f}^{2}$ scales approximately as 1/n

For the sake of completeness, even if not analyzed in this work, we can also mention that the noise due to the leakage current in the internal gate does not scale and increases with the total measurement time (see [12]). If the time of a single measurement is fixed, *i.e.*, if the total measurement time increases with number of measurements n:

• the white series noise and the 1/f series noise contributions scale as 1/n
• the noise due to the leakage current that fills the internal gate increases with n, *i.e.*, with the total measurement time.

From the above considerations, it follows that the repetitive non-destructive readout technique should be used when the 1/f noise is dominant. Given a system with defined input noise sources, it is in general possible to increase the readout time in order to make the white series noise negligible. Only at this point it is convenient to apply the RNDR processing scheme. Of course, in a real case, the total measurement time duration is limited by experimental constraints and by the leakage current that fills the internal gate of the devices, degrading the noise properties of the RNDR-DEPFET (see [12]).

On the other hand, the minimum time length of a single measurement is also limited in reality by technical constraints. In this case, in order to achieve a number of measurements n, sufficient to decrease the 1/f component to the desired value, it can be necessary to increase the total measurement time of the system, operating at a lower rate. In practice it is convenient to operate with flexible readout electronics [10,11] which allows one to change the time duration of the weighting function and the number of possible readout cycles. With this kind of tunability it is possible to trade speed for resolution with respect to the different experimental requirements, changing only the number n of multiple readouts and adjusting the weighting function duration accordingly.

We can evaluate in a more analytic and quantitative way the noise figure of a multiple signal processing accomplished by a filter implementing a triangular weighting function for every signal readout. We define an n-fold saw-tooth shaped weighting function, composed of n triangular weighing functions, as follows:

(5) $$\begin{array}{c} \text{Wf}\left(t\right)=\frac{1}{\tau }\overset{n}{\underset{k=1}{\sum }}\{\left[t-2\frac{\tau \left(k-1\right)}{n}\right]\cdot ℋ\left[t-2\frac{\tau \left(k-1\right)}{n}\right]-2[t-\frac{\tau \left(2k-1\right)}{n}]\\ \cdot ℋ\left[t-\frac{\tau \left(2k-1\right)}{n}\right]+\left[t-2\frac{\tau k}{n}\right]\cdot ℋ[t-\frac{\tau k}{n}]\} \end{array}$$

ℋ[t] represents the Heaviside function. The sum of the amplitude of the n individual signal readouts is normalized to one, *i.e.*, the n maxima Max[Wf(t)] are equal to 1/n. The shaping time τ is defined as the total available measurement time and does not depend on n.

The coefficients A_1_ and A_2_ can be calculated as follow:

(6) $$A_{1}=\int_{-\infty }^{+\infty }{\left[{\text{Wf}}^{\prime }\left(y\right)\right]}^{2}\text{dy}$$

(7) $$A_{2}=\int_{-\infty }^{+\infty }{\left[{\text{Wf}}^{\frac{\prime }{2}}\left(t\right)\right]}^{2}\text{dt}$$

where ${\text{Wf}}^{\frac{\prime }{2}}\left(y\right)$ is the derivative of order ½ of the weighting function and y = t/τ is the time normalized to the shaping time τ.

From Equations (5)–(7) it follows that A_1_ = 4 for every n, while A_2_ decreases as n increases. The A_2_ coefficients for different n values are reported in Table 1.

**Figure 7:**
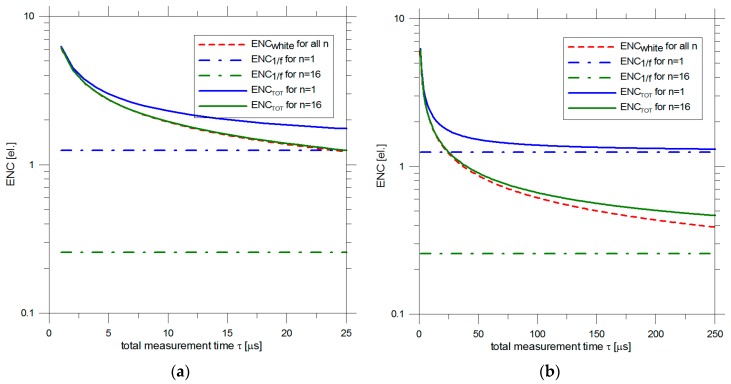
Calculated ENC components of a typical DEPFET processed by a triangular filter as a function of the total measurement time for two different number of readouts n = 1 and n = 16 for (**a**) fast readout and (**b**) slow readout.

As an example we can consider the measured noise power spectral density of an existing prototype DEPFET [17]. Typical physical noise sources values are: *a* = 1.5 × 10^−16^ V^2^/Hz and *af* = 4.5 × 10^−12^ V^2^. Table 1 reports the ENC component due to the 1/f noise for different number of readouts n, considering an equivalent input capacitance of the DEPFET of 40 fF. These values are independent from the total measurement time (shaping time) τ. Figure 5 shows the different ENC components as a function of τ for the cases n = 1 and n = 16. As stated before, ENC_white_ is the same for every n. From Figure 7a it is evident that for τ < 5–10 μs the overall ENC is dominated by the white noise and reading out the signal multiple times brings only a negligible improvement.

At shaping times longer than 10 μs, the multiple readout has a consistent impact on the overall noise. For τ = 200 μs (Figure 7b), for example, it is: ENC_TOT_ = 1.3 el. rms for n = 1 and ENC_TOT_ = 0.5 el. rms for n = 16.

In system where all the DEPFET pixels in one row of the sensor matrix are read out in parallel, e.g., in the focal plane of the MIXS instrument [18] with the ASTEROID ASIC [10], one can achieve sub-electron noise sensitivity reading out small matrices with a frame rate of some hundreds of Hz. With the mentioned measurement time per row of 200 μs, a 32 × 32 DEPFET array read out from two sides would provide a noise as low as 0.5 el. rms with a frame rate of about 300 Hz.

In a more sophisticated readout approach, e.g., the one adopted for the DEPFET sensor of the DSSC detector [19,20], one can use a dedicated readout channel for every pixel of the array. In this case the readout times needed for a single pixel and for the whole matrix are the same. It follows that a noise of 0.5 el rms is achievable with a frame rate of approximately 5 kHz. In this approach, which requires full parallel readout with an ASIC bump-bonded to the sensor, the frame rate is in principle not limited by the sensor size.

## 6. Experimental Evidence

**Figure 8:**
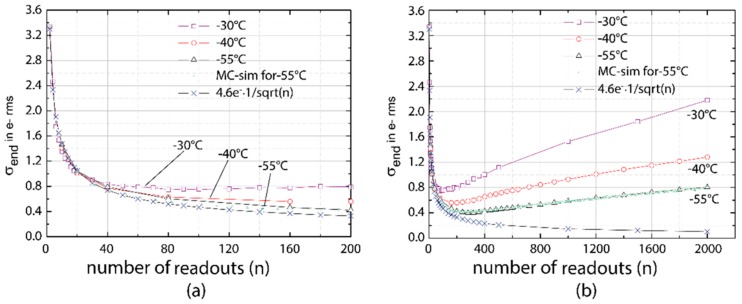
Experimental confirmation of the noise reduction due to multiple readouts using a fixed readout time of τ_SINGLE_ = 25.5 μs. For a single readout (n = 1) the readout noise for was σ_SINGLE_ = 4.6 e^−^ (**a**) Between 2 and 40 repetitive readouts the read noise follows approximately the expected behavior σ_end_ = σ_SINGLE_/sqrt(n) = 4.6 e^−^/sqrt(n); (**b**) For larger n values, the read noise is dominated by the leakage current and σ_end_ increases for higher n.

Several RNDR-DEPFET structures have been fabricated on a multi-project run and used to experimentally demonstrate the feasibility of the repetitive non-destructive readout of DEPFETs to beat the 1/f noise limit [8]. The impact of the leakage current on the achievable noise level, which for simplicity was neglected in the mathematical treatment, is illustrated in Figure 8. Measurements were done on one cell of a 4 × 4 mini-matrix with a cell size of 75 × 75 μm^2^. It shows the achievable noise σ_end_ (r.m.s.) for three different temperatures of −30 °C, −40 °C and −55 °C as a function of the number of readouts (n). The required time for a single readout τ_SINGLE_ was 25.5 μs, the total readout time increases linearly with n. Between n = 2 and n = 40 the noise follows the expected 1/sqrt(n) behavior. At the moderate temperature of −30 °C the total noise starts to increase again for n > 90 due to leakage current, which is collected in the internal gate during the multiple readouts and thus falsifies the number of stored signal electrons. For lower temperatures this turning point is shifted towards higher n and the curves approach more and more the expected 1/sqrt(n) behavior. Using a Monte-Carlo simulation and taking into account the measured leakage current of 0.02 e per ms and pixel at a temperature of −55 °C, the observed behavior can be well reproduced.

**Figure 9:**
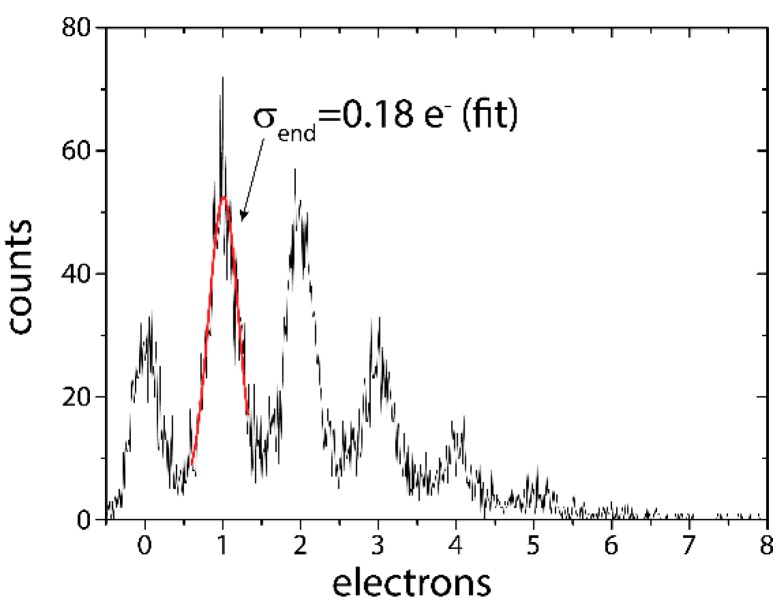
Single photon spectrum measured at low light intensity with a circular RNDR-DEPFET at a temperature of −55 °C. Due to the higher amplification the read noise of a single readout is only 3.1 e^−^ rms and a minimum noise of 0.18 e^−^ was obtained with only 300 readouts.

The lowest read noise so far was measured with a circular RNDR-DEPFET featuring a gate length of 5 μm and thus providing a significantly higher amplification compared to design used for the measurements shown in Figure 9. Due to the higher amplification a read noise of only 3.1 e^−^ r.m.s. was obtained for a single readout and consequently a noise of 0.18 e^−^ r.m.s. at a temperature of −55 °C was achieved after only 300 readout cycles with a readout time of 25.5 μs. In order to demonstrate the deep sub-electron resolution, the DEPFET pixel was illuminated by an optical laser (λ = 672 nm) with a very low intensity, generating only very few signal electrons in the pixel. The number of incident photons was subjected to Poissonian statistics, resulting in the spectrum shown in Figure 9.

**Figure 10:**
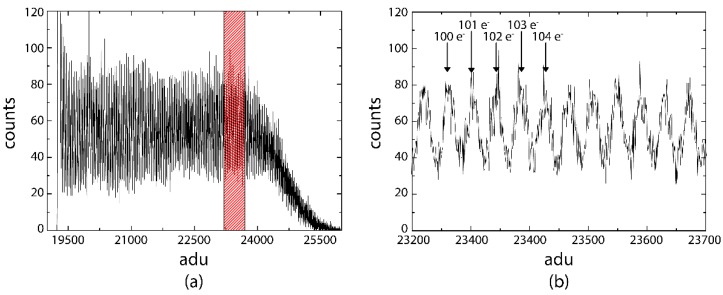
By continuously extending the exposure time of the DEPFET to a faint light source, a spectrum, which includes all photon numbers, can be induced. At an average of 120 electrons the exposure time was not increased any further, resulting in a Gaussian-shaped intensity drop for higher electron numbers (**a**). In the close-up view (**b**) of the red hatched area, well separated peaks can be seen and individual electrons can be counted even for high electron numbers.

In contrast to SiPMs, EMCCDs or other avalanche based detectors, the RNDR-DEPFET is a fully linear detector amplifier structure. In order to demonstrate this unique feature, the DEPFET pixel was illuminated with a faint light source so that during the integration time, on average a single photo electron was generated in the internal gate. Subsequently, the exposure time of the DEPFET detector to the light source was increased step by step and for each exposure time a spectrum was taken. By superimposing the various spectra for different exposure times a spectrum, as depicted in Figure 10a, was created. Up to 120 photons, a number which corresponds to the average for photos generated at the maximum exposure time, all photon numbers are equally represented. In the close-up (b) of the red hatched area, well separated peaks can be observed, and by counting the individual peaks, the number of incident photon can be precisely determined.

## 7. Summary and Outlook

We have described a sensor developed with the aim of being able to measure charge with sub-electron precision. This is useful for measuring the spectrum of low energy X-rays and especially for photon counting in the visible range. This sensor is based on the low-noise property of the DEPFET structure and its capability to read out the same signal charge repetitively. This RNDR readout method suppresses not only the white serial noise but also the low frequency 1/f noise that is normally independent of shaping conditions. A thorough theoretical treatment of noise for RNDR readout has been given. This treatment focusses on serial noise that is due to noise generated in the DEPFET transistors. Shot noise that is due to dark current in the sensor, however, was neglected. Its effect is an occasional increase in the charge that is shifted in and out of the internal gate of the DEPFET in all following read cycles. Thus, the measurement will result in non-integer multiples of the elementary charge. This problem is treated in [8]. Also neglected in the treatment are effects in the DEPFETs due to operation of the charge transfer mechanism.

Measurements on prototype RNDR DEPFET devices have shown excellent results. A sub-electron measurement precision of 0.18 electrons has been achieved with a moderate precision of 3.1 electrons for single readout. Furthermore distinction between n and n + 1 electrons is possible up to several hundred electrons. In the future, increased precision will be possible with improved noise for a single readout and more sophisticated readout electronics allowing for example readout of both DEPFETs of a pixel simultaneously. Furthermore, pixels can be read out in parallel, as is done for example in the DSSC project at the European XFEL, leading to an enormous frame rate, although at increased power consumption. The combination of RNDR-DEPFETs with an intermediate storage device [7] offers additional advantages such as simultaneous gating of all pixels and avoidance of sensitivity to signals during readout.

## Figures and Tables

**Table 1:** 1/f filter coefficients for a saw-tooth weighting functions composed of n triangles. The corresponding ENC due to the 1/f noise is calculated assuming *a_f_* = 4.5 × 10^−12^ V^2^/Hz and an DEPFET input capacitance of 40 fF.

Number of Readouts n	A_2_	ENC_1/f_
1	0.88254	1.25
2	0.38287	0.82
4	0.17038	0.55
8	0.07823	0.37
16	0.03695	0.25
32	0.01782	0.18
